# Pelvic Floor Muscle Training versus Functional Magnetic Stimulation for Stress Urinary Incontinence in Women: A Randomized Controlled Trial

**DOI:** 10.3390/jcm12093157

**Published:** 2023-04-27

**Authors:** Vilma Dudonienė, Indrė Kirklytė, Laura Žlibinaitė, Javier Jerez-Roig, Renata Rutkauskaitė

**Affiliations:** 1 Department of Health Promotion and Rehabilitation, Lithuanian Sports University, Sporto 6, LT-44221 Kaunas, Lithuania; 2 Department of Rehabilitation, Kauno Kolegija Higher Education Institution, Muitines 15, LT-44280 Kaunas, Lithuania; 3 Research Group on Methodology, Methods, Models and Outcomes of Health and Social Sciences (M3O), Faculty of Health Sciences and Welfare, Centre for Health and Social Care Research (CESS), University of Vic-Central University of Catalonia (UVic-UCC), 08500 Vic, Spain; 4 Department of Physical and Social Education, Lithuanian Sports University, Sporto 6, LT-44221 Kaunas, Lithuania

**Keywords:** stress incontinence, pelvic floor muscles, functional magnetic stimulation, exercise

## Abstract

Background: There is strong evidence that specific pelvic floor muscle training (PFMT) reduces stress urinary incontinence (SUI), but the application of functional magnetic stimulation (FMS) is still under discussion. Objective: To evaluate and compare the effects of FMS and PFMT on pelvic floor muscle function, urinary incontinence symptoms and quality of life (QoL) in women with SUI. Methods: A randomized controlled, parallel-group trial was executed in an outpatient physical medicine and rehabilitation centre. The study included 68 women and was fully completed by 48 women (*n* = 24 in each group) aged 29–49 years, with SUI, who were randomly assigned to PFMT and FMS groups. The symptoms of urinary incontinence and their impact on quality of life were assessed with two questionnaires: the International Consultation on Incontinence Questionnaire–Short Form (ICIQ-SF) and the Incontinence Impact Questionnaire–Short Form (IIQ-7). Perineometer (Pelvexiser) was used to measure the resting vaginal pressure, pelvic floor muscle (PFM) strength and endurance. All outcome measures were taken at baseline and after 6 weeks of interventions. Cohen’s effect size (*d*) was calculated. Results: A significant improvement (*p* < 0.05) of ICIQ-SF and IIQ-7 was observed in both groups with a high effect size in the PFMT group (*d* = 1.56 and *d* = 1.17, respectively) and the FMS group (*d* = 1.33 and *d* = 1.45, respectively). ICIQ-SF and IIQ-7 scores did not differ significantly between groups after the 6-week treatment period. Resting vaginal pressure, PFM strength and endurance increased (*p* < 0.05) in both groups with a medium (*d* = 0.52) to large (*d* = 1.56) effect size. Conclusion: No significant difference between groups was found in any measurement of perineometry. PFMT and FMS significantly improved SUI symptoms and the quality of life of the study participants. None of the applied interventions was superior to the other in the short-term effect.

## 1. Introduction

Urinary incontinence (UI) is a common health problem that negatively affects the physical and social quality of life [1] and economic and psychological well-being [2] both in women and men [3]. The main subtypes of UI are stress urinary incontinence (SUI), urgency and mixed. The prevalence of UI varies depending on different incontinence definitions and assessment methodologies used [4]. SUI is characterized by an involuntary loss of urine on physical effort or exertion or when coughing or sneezing [5]. SUI is the predominant UI type among adult women [6]. Major risk factors for SUI include female gender, older age, overweight or obesity, multiple births, menopause, dystocia, urinary tract infections, diabetes mellitus, chronic respiratory problems, lifestyle or high-intensity exercise [3,6,7,8]. Although vaginal delivery is considered a physiological way of delivery, it can also be associated with urinary and even faecal incontinence, because during prolonged vaginal delivery, the pelvic floor muscles can be pressed and get overstretched [9,10]. Barca et al. [11] state that vaginal delivery is directly related to the appearance of pelvic floor disorders, mainly UI, pelvic organ prolapse and anal incontinence.

Many women with SUI symptoms report frustration, anxiety, depression, reduction of self-esteem, feeling of shame and problems in sexual function [3,12,13]. Studies demonstrate that even mild urinary leakage significantly reduces the quality of life QoL [14,15]. A systematic review and meta-analysis including 23 studies and 24,983 participants showed that the presence of UI was significantly associated with poor QoL [16].

Treatment interventions for SUI can include non-surgical options, such as pelvic floor muscle training (PFMT), electrostimulation, magnetic stimulation, vibration and biofeedback [3,12] and radiofrequency or laser therapy [17]. However surgical treatment aims to support the urethra or increase bladder capacity [12]; PFMT is considered the first-line approach to treating SUI [18,19]. Fitz et al. [20] and Felicíssimo et al. [21] reported equal benefits of supervised (outpatient) and unsupervised (home programs) PFMT for improving female SUI, and this may be considered an option for self-management strategy [22]. Specific PFMT exercises, called Kegels, are proven to be effective for female UI and pelvic organ prolapse and have been recommended as the initial therapeutic option [23], but the training needs proper instructions and close follow-up to be effective [24].

Despite the strong evidence of the effectiveness of PFMT for the treatment of SUI [25] there seems to be increasing interest in using functional magnetic stimulation (FMS) [26]. Magnetic stimulation, sometimes called extracorporeal magnetic innervation, is described as a pulsed magnetic technology developed for the transmission of nerve impulses. The aim of FMS is to cause pelvic floor muscle (PFM) contractions by producing pulsing magnetic fields [27]. FMS is a non-invasive and safe treatment for SUI [28]. The setting (patient sitting on a chair with clothes) and the lack of direct activation of skin sensory receptors and C-fibers make this procedure comfortable and painless [17]. Magnetic stimulation procedures have been observed to reduce SUI symptoms without any side effects [29] by stimulating both peripheral and central nerves and resulting in sacral S2-S4 roots neuromodulation [30], thus causing muscle contraction [31].

The efficacy of incontinence treatment is frequently evaluated by patient-reported outcomes using questionnaires. The most frequently used subjective measure is ICIQ-SF [32] and it should be sensitive enough to detect the smallest change that is considered clinically important [33].

There have been many articles published in 5 recent years [17,23,26,28,29,31] discussing the benefits of using FMS in the management of UI. Peng et al. [34] state that “magnetic stimulation leads to an improvement in SUI without any reported safety concerns and an improvement in patient quality of life”, but the authors agree on the uncertainty of the long-term effect of this technique. The aim of this research was to evaluate the effect of pelvic floor muscle training and functional magnetic stimulation performed with Magneto STYM device on pelvic floor muscle strength, endurance, resting vaginal pressure, SUI symptoms and quality of life in women.

## 2. Materials and Methods

### 2.1. Ethical Approval

The study protocol was approved by the Bioethics Committee (No. MNL-KIN(M)-2021-404) of the Lithuanian Sports University and registered at ClinicalTrials.gov (Identifier: NCT05721807; accessed on 19 January 2023). All participants were informed in detail of the purpose and procedures of the study, and they signed an informed consent form. The study was conducted in accordance with the Declaration of Helsinki Ethical Principles and Good Clinical Practices.

### 2.2. Study Design

This parallel-designed trial was conducted in an outpatient Rehabilitation clinic in Vilnius, Lithuania. Women who were diagnosed with SUI by a urogynecologist were invited to participate in the study. Those women who agreed to participate in the study were evaluated by a urogynecologist who performed the interview and physical examination. As recommended by the 6th International Consultation on Incontinence [35], the urogynecological examination included abdominal, pelvic and perineal examinations; women were asked to perform a “stress test” (cough and strain to detect leakage). After evaluation, the patients were randomly assigned into the two groups using a list of previously generated blinded intervention codes and an automatic assignment system (random.org, accessed on 7 July 2021) to conceal the allocation. After that, women were prescribed physiotherapy interventions. Physiotherapists were blinded to patients’ pre- and post-assessment results. The urogynecologist did not know which group the women were assigned to. Patients who agreed to participate in the study were aware of the physiotherapy they received. This randomized controlled trial allocated women with SUI to either a 6-week supervised outpatient pelvic floor muscle training (PFMT) group or a functional magnetic stimulation (FMS) group. Sample size calculation was performed using statistical software G*Power 3.1.9.2 with a power of 80%, a significance level of 0.05 and an effect size of 0.50. The estimated desired sample size was 34.

**Figure 1:**
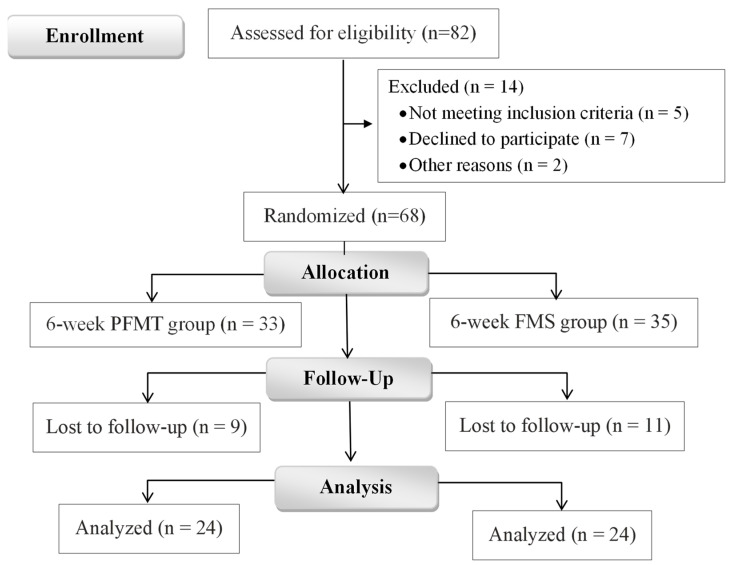
CONSORT flow chart.

The study protocol was prepared following the CONSORT guidelines (Figure 1).

### 2.3. Participants

Women with only SUI participated in the study. The inclusion criteria were having an age between 29 and 49 years, complaints of episodes of SUI for at least 4 weeks and women who had at least one vaginal delivery. Subjects were excluded if they were pregnant or were diagnosed with vaginismus, urinary tract infections, cancer, epilepsy, pelvic organ prolapse greater than stage I, skin diseases, had undergone previous pelvic floor surgeries or had a heart stimulator or a metal implant and were unable to contract the PFM. After the gynecological evaluation, 82 participants with SUI were selected, but 14 were excluded because they did not meet the inclusion criteria. Sixty-eight women (in group PFMT *n* = 35, and in group FMS—*n* = 33) agreed to participate in the study and only forty-eight (70.6%) fully completed the interventions.

At baseline, there were no significant differences between the groups in terms of age, weight, height, body mass index and physical activity (Table 1).

### 2.4. Outcome Measures

*ICIQ–SF—International Consultation on Incontinence Questionnaire–Short Form*. This questionnaire is short and easy to use in a clinical setting, assessing the severity of UI and its impact on QoL [36]. The questionnaire consists of four items and the overall score ranges from 0 to 21, with greater values indicating increased symptom severity: 0—no symptoms of UI, 1–5 scores—mild symptoms of UI, 6–12 scores—moderate symptoms of UI, 13–18 scores—severe symptoms of UI and 19–21 scores—very severe symptoms of UI [37].

*IIQ-7—Incontinence Impact Questionnaire*. The Incontinence Impact Questionnaire short version (IIQ-7) is useful to quickly quantify the UI-related life impact [38]. It consists of seven items and the total score ranges from 0 to 100 [39].

*Perineometry*. This was conducted with the pressure perineometer Pelvexiser, which consists of an air-filled vaginal balloon (75 mm length and 28 mm in diameter) connected to a high-precision pressure transducer (Wolfram Haboeck Co., Vienna, Austria). Perineometry is a simple, minimally invasive, low cost and reliable quantitative method [10]. Women were tested in a supine position with the knees bent and legs slightly apart and were instructed to perform PFM contraction without any movement of the pelvis or visible contraction of the gluteal, hip or abdominal muscles [40]. The testing procedure was explained to all patients individually, and they were asked to show their best results but were not motivated during testing. Three measurements were performed with 2 min rest between them.

1. The resting vaginal pressure (mmHg) was registered when a perineometer was inserted into the vagina without contracting PFM.
2. PFM strength was calculated as the mean of three isolated maximal voluntary contractions (mmHg). The patient was asked to contract their pelvic floor muscles to a maximum without holding their breath three times with a 5 s rest between trials.
3. PFM endurance was calculated as the mean of three endurance trials (s). Participants were asked to hold an isolated maximal voluntary PFM contraction for as long as they could without holding their breath. The trial was stopped when the squeeze pressure dropped by 2 mm. There was a 10 s rest between the three trials.

### 2.5. Interventions

Subjects of both groups completed 12 individual training sessions lasting for 6 weeks (two times a week). During the first session subjects were explained the anatomy and function of PFM and how to correctly perform contraction.

*Pelvic floor muscle training*. Women in the PFMT group received 12 sessions (30-minute duration) that followed a specific exercise program. The PFMT program consisted of two parts (Table 1). The exercise sessions were organised individually under the supervision of an experienced physiotherapist. From sessions 1 to 6, six exercises were performed focusing on slow- and fast-twitch fibers of the pelvic floor muscles (strength, endurance, power and relaxation), diaphragmatic breathing, transversus abdominis contraction and strengthening of thighs, buttocks and core muscles. From sessions 7 to 12, another five exercises were added. These exercises included progression from gravity-eliminated body positions to antigravity, exercise “elevator”, and lumbo-pelvic stability training. The exercises were performed in 2 sets of 10 repetitions each with 30–60 s rest intervals in between. While exercising, attention was paid to correct breathing patterns. Body positions were changed progressively from supine to side-lying, sitting, and quadruped [41]. The intensity level was customized for each participant and based on their functional capacity.

**Figure 2:**
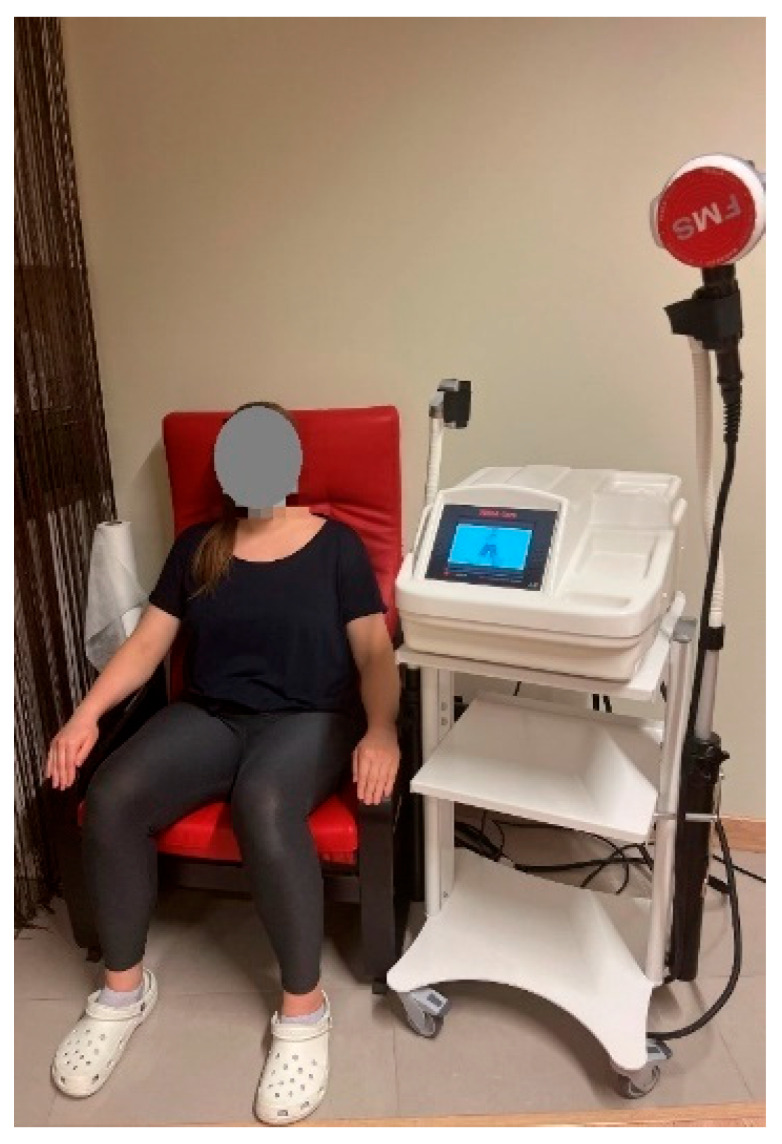
FMS procedure with the Magneto STYM device.

*Functional magnetic stimulation*. FMS was performed with the Magneto STYM device (Iskra Medical d.o.o. Stegne 23, 1000 Ljubijana, Slovenia). It is a chair developed for the treatment of UI. The magnetic coil was positioned at the bottom of the chair. During the treatment, each subject was instructed to sit on a chair so that the perineum was in the centre of the coil to feel the contraction of PFM (Figure 2).

When applying FMS, the SUI program “P2 stress” was chosen. Stimulation frequency for the first 20 min was set at 35 Hz (modulation—rising amplitude from 0 to maximum per second; total wave duration 12 s, active time 6 s, pause time 6 s) [31]. For the last 10 min, stimulation frequency was changed to 5 Hz (modulation and wave duration remained the same) (Table 2). The total duration of the procedure was 30 min.

### 2.6. Statistical Analysis

The data were tested for normal distribution using the Shapiro–Wilk test; all data were found to be normally distributed. The two groups were compared at baseline with the Student’s *t*-test for continuous variables and the chi-square test for categorical variables. Values are reported as mean and standard deviation. A mixed design analysis of variance (ANOVA) was used to determine the effects of treatments on selected outcome measures. Values are reported as mean, standard deviation and percentage. The level of significance was set at *p* < 0.05. Data were analyzed with Cohen’s d effect sizes to examine the magnitude of change in outcomes following the intervention, and the effect size was interpreted as follows: 0.0–0.2 small effect, 0.5–0.7 medium and 0.8–2.0 large effect. The data obtained were analyzed using IBM SPSS Statistics (version 26.0, IBM Corp., Armonk, NY, USA).

## 3. Results

Sixty-eight participants were randomized and forty-eight completed the study (dropout rate 29.41%). There were no adverse effects reported by patients post interventions. The results of the severity of involuntary urine loss measured by ICIQ-SF and IIQ-7 are presented in Table 3. In both groups, the severity of UI symptoms was indicated as moderate to severe before the interventions. A significant decrease in the total ICIQ-SF score was observed both in the PFMT group (*p* < 0.001; Cohen’s *d* = 1.56) and in the FMS group (*p* < 0.001; Cohen’s *d* = 1.33). Herewith, the total IIQ-7 score significantly decreased after the PFMT (*p* < 0.001; Cohen’s *d* = 1.17) and FMS (*p* < 0.001; Cohen’s *d* = 1.45) interventions. However, ICIQ-SF and IIQ-7 scores did not differ significantly between groups after the 6-week treatment period.

Data for the perineometry are presented in Table 3. The resting vaginal pressure significantly improved in both the PFMT group (*p* < 0.001; Cohen’s *d* = 1.20) and the FMS group (*p* < 0.001; Cohen’s *d* = 1.54). Moreover, the PFM strength significantly increased in both the PFMT group (*p* < 0.001; Cohen’s *d* = 0.85) and in the FMS group (*p* < 0.05; Cohen’s *d* = 0.72). The significant improvement in the PFM endurance was observed after the PFMT (*p* < 0.05; Cohen’s *d* = 0.62) and FMS interventions (*p* < 0.05; Cohen’s *d* = 0.52). No significant difference between the groups was found in any measurement of perineometry.

**Figure 3:**
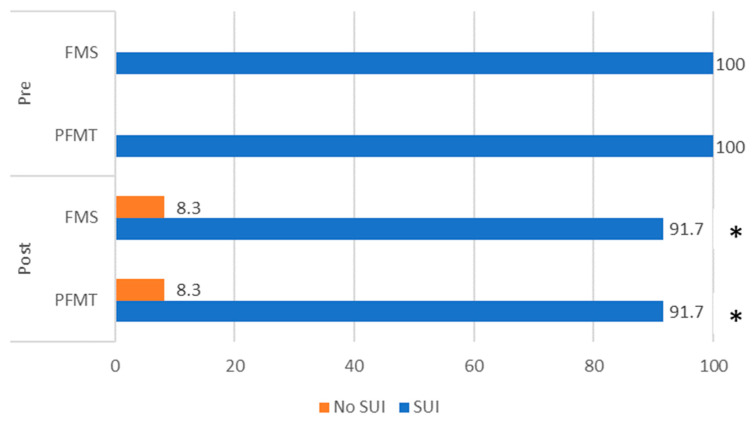
Distribution of study participants according to complaints of SUI pre- and post-interventions. Note: *—*p* < 0.05 between pre- and post-interventions within groups. PFM, pelvic floor muscle; PFMT, pelvic floor muscle training; FMS, functional magnetic stimulation.

The results of ICIQ-SF items are presented in Figure 3. Before the interventions, 100% of women complained of UI with sneezing or coughing and physical exertion. The number of participants with SUI reduced after the interventions—8.3% of study participants in both groups reported that symptoms of UI were eliminated after treatments (Figure 3).

**Figure 4:**
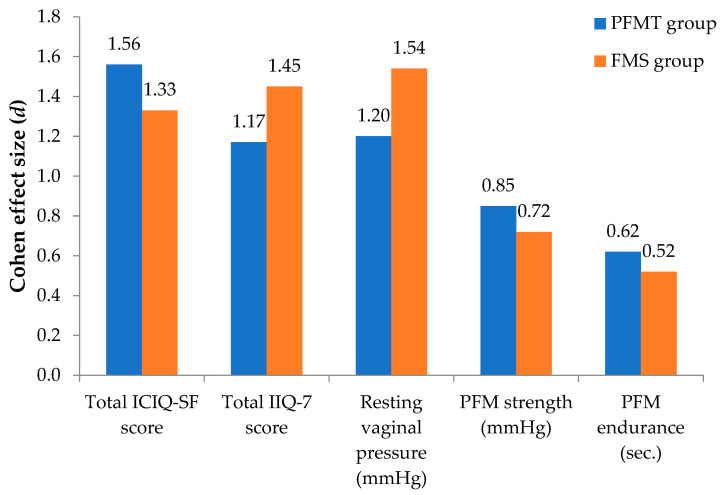
Comparison of Cohen’s d effect size values between the groups. Note: ICIQ-SF, International Consultation on Incontinence Questionnaire–Short Form; IIQ-7, Incontinence Impact Questionnaire; PFM, pelvic floor muscle; PFMT, pelvic floor muscle training; FMS, functional magnetic stimulation.

As shown in Figure 4 the effect sizes of both interventions on outcome measures were similar. The large effect size was determined with total ICQ-SF and IIQ-7 scores and resting vaginal pressure in both groups and on PFM strength in the PFMT group. The medium effect size was determined with PFM endurance in both groups and on PFM strength in the FMS group.

## 4. Discussion

This preliminary experimental study aimed to investigate the effectiveness of FMS and PFMT on pelvic floor muscle function, urinary incontinence symptoms and the quality of life in women with SUI. Our results showed that both groups significantly improved all these outcomes, with high effect sizes and no superiority for any group.

UI is recognized as a major health problem especially among women [42], negatively affecting their quality of life [1,3,12,13]. Although older age and obesity or being overweight are considered risk factors for UI, our study participants were relatively young (mean age 38.92 ± 6.49 years) and non-obese (mean BMI 24.78 ± 3.43 kg/m^2^) but had given at least one birth vaginally. The improvement in the severity of involuntary urine loss measured by ICIQ–SF and IIQ-7 was the primary outcome measure showing the effect of interventions used in our study. QoL scores are considered to be the most important indicator when evaluating treatment [42]. After both interventions used in our study QoL in women with SUI greatly improved with a high effect size. All the females in this study complained of involuntary urine loss during coughing, sneezing or physical exertion before interventions. Results of our study showed that interventions lasting for 6 weeks eliminated SUI symptoms only in 8.3% of women, nevertheless, the quality of life of all subjects improved significantly. From this, we could suggest prolonging the treatment duration. Ongoing research not only should last longer but evaluate the effects of different magnetic stimulation protocols, i.e., different stimulation frequencies, modulation and wave duration.

As recommended in the scientific literature [20,21], we applied supervised interventions to our patients. The resting vaginal pressure as well as PFM strength and endurance improved significantly after both interventions. PFMT programs are proven to be effective in treating SUI [18,19,25,43] with and even without biofeedback [44]. The effectiveness of interventions depends on the improvement in QoL and PFM strength [44]. García-Sánchez with co-authors [45] showed, in the meta-analysis conducted in 2019, that PFMT did not depend on the protocol used in the study and was effective regardless of the women’s age (under 53 and over 53 years old). Different training protocols resulted in decreased urine loss in females diagnosed with SUI. The authors suggested intervention programs to last 6–12 weeks. To reach a large effect size, more than 3 sessions per week with a length of one session lasting more than 45 min are recommended [45].

Pulsed magnetic stimulation is a non-invasive treatment in which patients can undergo a procedure while fully clothed [46]. The changing magnetic field leads to pelvic floor nerve stimulation and repetitive PFM contractions [47] similar to PFMT. Lim et al. [28] found that pulsed magnetic stimulation applied for SUI in 35 women involved in 2 sessions per week for 2 months (16 sessions, 20 min each with 50 Hz in an 8 s on 4 s off pulsing manner) improved physical, social and psychological aspects of QoL [28]. In our study, patients received 12 physiotherapy sessions applied two times a week for 6 weeks, but the duration of every session was longer—30 min—and the stimulation frequency in our study was lower. Yamanishi et al. [29] used the stimulation of 50 Hz in 5 s on/5 s off cycles for 10 weeks with one session lasting 20 min and found magnetic stimulation to be effective and safe in the treatment of SUI in 18 women. Weber-Rajek et al. [48] found that even a 4-week duration PFMT, as well as magnetic innervation, were effective treatment methods for SUI in women. In addition, Sun et al. [49] demonstrated that the IIQ-7 scores were statistically significantly reduced even after 8 sessions of extracorporeal magnetic innervation. For women undergoing nonsurgical treatments for incontinence, a reduction of 4 points in ICIQ-SF is perceived as clinically meaningful [30]. In our study, the reduction of ICIQ-SF scores was higher than 4 points and can be considered as clinically important. Furthermore, Vadala et al. [31] concluded that FMS with the Magneto STYM device used twice weekly for 3 weeks had significant advantages on 20 patients with SUI aged 38–82 years without any adverse effects.

A high percentage of females can not voluntarily control PFM [20]; therefore, physiotherapists working in clinical practice must find ways how to educate women to contract their PFM or to search for other effective and evidence-based treatment methods. Some researchers [50] recommend conservative UI treatment with FMS for patients who are not motivated to perform regular PFM exercises.

Even though magnetic stimulation is widely used in the treatment of UI [23,28,29,30,31,34,46,47,48,49,50], it is necessary to evaluate the indications and contraindications of this technique [51].

The main limitations of our study were a relatively small sample size and a lack of follow-up to assess the long-term effects of the interventions. On the other hand, the topic is very sensitive and intimate; many women uncover that they have problems and refuse to participate in research. The age range of the subjects in this study was quite wide (20 years), which could have influenced the results negatively. Another weakness of our study is that the data analysis did not consider the level of physical activity of women, which is presented in the characteristics of the subjects. In addition, the time period after childbirth, the number of deliveries and the satisfaction with treatment should be considered. In the future, larger studies involving long-term outcomes and sham FMS interventions should be planned, and a control group with no interventions could be involved. The strengths of our study are that both groups were homogeneous. The interventions were conducted in parallel and lasted for 6 weeks; therefore, the weather and other environmental factors were the same for the subjects and had the same influence on UI symptoms. Furthermore, our study was double-blinded, as the urogynecologist was not provided with the groups that patients were assigned to, and physiotherapists were not introduced to the results of patients’ assessment pre-interventions.

Results of our study showed that physiotherapists working in clinical settings can prescribe PFMT programs as well as FMS interventions, because of the high and similar effect sizes and the safety of the treatments.

## 5. Conclusions

A six-week PFMT program, as well as low-frequency FMS intervention performed with a Magneto STYM device, improved pelvic floor muscle strength, endurance, resting vaginal pressure, UI symptoms and the quality of life in relatively young women with SUI. None of the applied programs were superior to the other in the short-term effect. Both interventions were safe and well tolerated by the study participants. Further research is needed to investigate the long-term effects of FMS.

## Figures and Tables

**Table 1:** Characteristics of the participants.

	PFMT Group (*n* = 24) Mean ± SD	FMS Group (*n* = 24) Mean ± SD	*p *between Groups (Student’s *t* Test)
Age (years)	37.58 ± 5.86	40.25 ± 6.49	0.142
Weight (kg)	69.79 ± 8.14	74.25 ± 10.64	0.062
Height (cm)	168.00 ± 4.00	168.50 ± 5.23	0.712
Body mass index (kg/m^2^)	23.44 ± 3.01	26.13 ± 3.43	0.371
Physically active (%)	66.67%	75%	0.535
Frequency of physical activity (days/week)	2.17 ± 2.28	2.50 ± 2.02	0.594

Note: PFMT—pelvic floor muscle training; FMS—functional magnetic stimulation.

**Table 2:** Description of the interventions.

**Functional Magnetic Stimulation**	**Frequency of Stimulation**	**Time**	**Active Time**	**Pause Time**	**Duration of Session**	**Number of Sessions**
	35 Hz	12 s	6 s	6 s	20 min.	12 sessions
	5 Hz	12 s	6 s	6 s	10 min	
**Pelvic Floor Muscle Training**	**Step 1**	**Step 2**			**Duration**	**Number of Sessions**
	Session 1–6	Session 7–12			30 min.	12 sessions
	6 exercises	6 + 5 exercises				

**Table 3:** Comparison of outcome measures between groups in pre- and post-intervention assessments.

Measurement	Group	Pre Mean ± SD	Post Mean ± SD	Mean Difference	*p* Inter- Group
Total ICIQ-SF score	PFMT	11.00 ± 3.68	6.33 ± 3.07	4.67 ± 2.99 **	0.509
	FMS	9.17 ± 3.33	5.08 ± 2.45	4.08 ± 3.08 **	
Total IIQ-7 score	PFMT	33.25 ± 23.25	10.75 ± 11.54	22.50 ± 19.26 **	0.699
	FMS	30.42 ± 14.36	9.33 ± 3.62	20.58 ± 14.57 **	
Resting vaginal pressure (mmHg)	PFMT	4.67 ± 1.79	6.67 ± 1.27	2.00 ± 1.67 **	0.089
	FMS	5.58 ± 2.19	6.92 ± 1.74	1.33 ± 0.87 **	
PFM strength (mmHg)	PFMT	11.59 ± 5.72	15.36 ± 5.88	3.77 ± 4.43 **	0.458
	FMS	15.08 ± 7.59	17.94 ± 8.32	2.86 ± 3.96 *	
PFM endurance (s)	PFMT	10.54 ± 9.28	18.97 ± 15.60	8.43 ± 13.55 *	0.661
	FMS	11.03 ± 11.52	17.79 ± 22.33	6.76 ± 12.69 *	

Note: *—*p* < 0.05 between pre- and post-interventions within groups. **—*p* < 0.001 between pre- and post-interventions within groups. Abbreviations: PFM, pelvic floor muscle; PFMT, pelvic floor muscle training; FMS, functional magnetic stimulation.

## Data Availability

Not applicable.
